# The Impact of Telemedicine and Remote Patient Monitoring on Healthcare Delivery: A Comprehensive Evaluation

**DOI:** 10.7759/cureus.55534

**Published:** 2024-03-04

**Authors:** Vijaya Krishna Prasad Vudathaneni, Rama Brahmam Lanke, Manasi Chinnadurai Mudaliyar, Kalikrishna Varaprasad Movva, Lakshmi Mounika Kalluri, Ramanarayana Boyapati

**Affiliations:** 1 Internal Medicine, Albert Einstein College of Medicine, Bronx, USA; 2 Dentistry, Familia Dental, Midland, USA; 3 Dentistry, Private Practice, Midland, USA; 4 Anaesthesiology, Aster Ramesh Hospitals, Guntur, IND; 5 Prosthodontics, Sibar Institute of Dental Sciences, Guntur, IND; 6 Periodontology, Sibar Institute of Dental Sciences, Guntur, IND

**Keywords:** healthcare accessibility, patient satisfaction, economic impact, patient outcomes, chronic conditions, healthcare delivery, remote patient monitoring, telemedicine

## Abstract

Background

Telemedicine and remote patient monitoring have emerged as transformative solutions in contemporary healthcare. This study aimed to conduct a comprehensive evaluation of the impact of these technologies on healthcare delivery, focusing on patient outcomes, economic parameters, and overall satisfaction.

Methods

A prospective observational study was conducted in various healthcare facilities, involving 186 participants with chronic diseases. Inclusion criteria included patients actively using telemedicine services. Data collection methods included surveys, interviews, and review of medical records, focusing on patient demographics, clinical outcomes, and economic parameters. The intervention involved a seamless integration of telemedicine technologies into the existing health system.

Results

Primary outcomes revealed significant improvements in patient health, including a decrease in disease-specific markers (mean reduction of 12,000 to 11,000, p = 0.002), a substantial reduction in severity of symptoms (mean reduction from 3,500 to 2,500, p < 0.001), and a general improvement in health status (mean increase from 7,200 to 8,500, p < 0.001). The savings in healthcare costs were evident, with direct costs decreasing from 25,000 to 12,000 (p < 0.001) and indirect costs decreasing from <10,000 to <5,000 (p = 0.004). Secondary results demonstrated increased patient satisfaction with communication (increase from 80% to 95%, p < 0.001) and convenience of services (increase from 75% to 90%, p < 0.001). Patient satisfaction also increased significantly (from 80% to 95%, p < 0.001). Accessibility to healthcare services improved, with a reduction in geographic barriers (increase from 65% to 90%, p < 0.001) and a decrease in the frequency of healthcare utilization (decrease from 2.5 to 1.5, p < 0.001).

Conclusion

The study provides robust evidence of the positive impact of telemedicine and remote patient monitoring on healthcare delivery. Significant improvements in patient outcomes, coupled with substantial cost savings and increased satisfaction levels, underscore the transformative potential of these technologies.

## Introduction

The landscape of healthcare delivery has seen transformative changes in recent years, with advances in technology playing a pivotal role in shaping the future of patient care [1]. Among these technological innovations, telemedicine and remote patient monitoring have emerged as critical components, offering unprecedented opportunities to improve accessibility, improve patient outcomes, and optimize healthcare resource utilization [2].

The global healthcare landscape faces a wide range of challenges, including rising healthcare costs, disparities in access to medical services, and the burden of chronic diseases [3]. Telemedicine and remote patient monitoring hold immense promise in addressing these challenges by providing cost-effective solutions, improving healthcare accessibility, and empowering individuals to actively manage their health [4]. In particular, in regions with limited access to healthcare facilities, telemedicine emerges as a crucial tool to bridge the gaps in healthcare delivery, offering remote consultations and continuous monitoring [5].

Furthermore, the COVID-19 pandemic has accelerated the adoption of telemedicine, focusing on its role in ensuring continuity of care while minimizing the risk of virus transmission [6]. The unprecedented demand for virtual healthcare services during the pandemic underscored the resilience and adaptability of telemedicine to maintain essential healthcare functions [7].

Although the potential benefits of telemedicine and remote patient monitoring are evident, a rigorous evaluation through empirical studies is essential to validate their impact on healthcare delivery. This study aims to comprehensively assess the implications of these technologies on patient health outcomes, healthcare costs, and overall quality of care. By examining a diverse pool of participants in multiple healthcare facilities, the study seeks to provide nuanced insights into the real-world effectiveness and challenges associated with the integration of telemedicine and remote patient monitoring.

The primary objectives of the study include evaluating the impact of telemedicine and remote patient monitoring on patient health outcomes, assessing the economic implications in terms of healthcare care cost savings, and gauging the satisfaction levels of patients and healthcare providers. In addition, the study aims to explore changes in the accessibility of healthcare services and address potential challenges associated with the adoption of these technologies.

## Materials and methods

Study design

The research was meticulously designed as a prospective observational study, spanning multiple healthcare facilities to ensure a diverse and representative group of participants. The study included a total of 186 participants, covering a variety of demographic and healthcare settings. This study sought to comprehensively assess and understand the impact of telemedicine and remote patient monitoring on the delivery of healthcare services.

Ethical considerations

The study adhered to ethical standards with complete compliance, prioritizing the well-being and rights of the participants. Informed consent was obtained from all participants, ensuring transparency and respect for individual autonomy. The study protocol, including the consent process, was reviewed and approved by the relevant Institutional Review Board (IRB) IEC/SIDS/18/2022.

Participants

The inclusion criteria were carefully designed to involve patients grappling with chronic diseases, ensuring a focus on populations that could potentially benefit significantly from telemedicine and remote patient monitoring. In addition, the study actively engaged healthcare providers who were proficient in using telemedicine services in their practice. This dual-participant approach aimed to capture a holistic perspective, considering both the recipients of healthcare services and the providers who provide them. The exclusion criteria were methodically defined, excluding individuals who were unable to provide informed consent, thus upholding ethical standards and ensuring participant autonomy in the study.

Data collection

The data collection process was thoughtfully designed to gather comprehensive and nuanced information, employing a combination of methodologies to ensure a well-rounded understanding of the impact of telemedicine and remote patient monitoring. Rigorous validation measures were incorporated to enhance the reliability and credibility of the collected data.

Surveys

A structured survey instrument, developed based on established scales and validated questionnaires in the literature, was used to obtain quantitative data from the participants. Before implementation, the survey instrument underwent pilot testing with a small group of participants to assess clarity, relevance, and comprehensibility. Feedback from the pilot phase was carefully considered, leading to refinements of the survey questions to ensure validity and reliability.

Interviews

The interview guide was constructed using established qualitative research principles. A pilot phase was conducted with a sample of participants to validate the interview questions for clarity and relevance. Additionally, an inter-rater reliability check was performed by involving multiple interviewers to ensure consistency in data collection. Regular debriefing sessions were held among the interviewees to discuss emerging themes, enhancing the reliability of qualitative data interpretation.

Medical Records Review

The review process of medical records adhered to standardized protocols to ensure consistency and precision. A random sample of records was independently reviewed by two trained researchers, and any discrepancies were resolved by consensus. To validate the reliability of data extraction, a third reviewer cross-checked a subset of records. This iterative process aimed to minimize potential errors and biases in data extraction, enhancing the general validity of clinical information.

Variables of Interest

Demographic data for the patients, clinical outcomes, and economic parameters were defined with precision to align with widely accepted metrics. The study team participated in regular calibration sessions to ensure uniform interpretation and application of these variables, contributing to the internal validity of the study.

Intervention

The integration of telemedicine and remote patient monitoring into the healthcare delivery system was a critical component of the study, designed to address the multifaceted challenges of contemporary healthcare. This integration was not merely about deploying new technologies but about transforming the way healthcare is delivered to make it more accessible, efficient, and patient-centered.

Selection and Customization of Technologies

A wide array of telemedicine technologies was carefully evaluated to select those best suited for the study's objectives. This selection process considered factors such as user-friendliness, technical reliability, and compatibility with existing healthcare systems. Custom software solutions were developed in some cases to ensure seamless integration with electronic health record (EHR) systems, facilitating a smooth workflow for healthcare providers. The chosen technologies supported a range of functionalities, from video conferencing for virtual consultations to wearable devices for continuous monitoring of vital signs, allowing for a comprehensive approach to patient care.

Implementation and Training

Implementing these technologies required a detailed plan that involved not only the technical setup but also extensive training for both healthcare providers and patients. Training sessions were designed to be interactive, covering the operational aspects of the technology, best practices for virtual consultations, and strategies for effective patient engagement remotely. For patients, the training focused on how to use the devices and platforms, interpret their health data, and communicate effectively with their healthcare providers. This education aimed to empower patients, enabling them to take an active role in their healthcare management.

Pilot Testing and Feedback Loops

Before full-scale deployment, pilot tests were conducted in select healthcare settings to identify potential issues and gather feedback from early users. These tests were crucial for assessing the practicality of telemedicine solutions in real-world scenarios. Feedback from both patients and healthcare providers during this phase led to refinements in the technology and its application, ensuring that the final rollout would be as effective and user-friendly as possible.

Integration With Healthcare Processes

Telemedicine and remote patient monitoring technologies were integrated into the healthcare providers' existing workflows in a manner that complemented and enhanced traditional care delivery methods. This included establishing protocols for when and how to conduct virtual consultations, integrating patient-generated health data into clinical decision-making processes, and developing new communication strategies to maintain patient engagement. The aim was to create a hybrid model of care that leveraged the best of both in-person and virtual healthcare services.

Ongoing Support and Adaptation

Recognizing the dynamic nature of technology and healthcare needs, the study included provisions for ongoing technical support and periodic reviews of telemedicine implementations. This support structure ensured that healthcare providers and patients could resolve technical issues swiftly, minimizing disruptions to care. Furthermore, the study's design allowed for adaptations to the telemedicine solutions based on evolving healthcare practices, patient needs, and technological advancements, ensuring that the telemedicine integration remained relevant and effective over time.

In essence, the integration of telemedicine and remote patient monitoring was a comprehensive effort that extended beyond the mere deployment of technology. It was about creating a sustainable, adaptable, and patient-centric healthcare delivery model that could meet the challenges of modern healthcare and pave the way for future innovations.

Outcome measures

The study used a comprehensive set of outcome measures to holistically assess the impact of telemedicine and remote patient monitoring on healthcare delivery. Primary and secondary outcomes were meticulously selected to capture a multifaceted understanding of the intervention's effectiveness.

Primary Outcomes

Patient health improvements: The evaluation of patient health improvements was intricately designed, incorporating a range of clinical indicators specific to chronic conditions. Objective measures included disease-specific markers, severity of symptoms, and general health status. These metrics were tracked longitudinally, allowing for a detailed analysis of any changes in health outcomes throughout the study. Patient-reported results obtained through surveys and interviews further enriched the understanding of subjective health improvements.

Healthcare cost savings: The economic impact of telemedicine and remote patient monitoring was quantified by assessing healthcare cost savings. This involved a meticulous analysis of direct costs related to hospital visits, emergency room admissions, and medication expenses. Indirect costs, such as productivity loss and transportation expenses for patients, were also considered. A comparative analysis of healthcare utilization and associated costs before and after the intervention provided a robust assessment of financial implications.

Secondary Outcomes

Patient satisfaction: Patient satisfaction was evaluated through a combination of quantitative surveys and qualitative interviews. The surveys included standardized satisfaction scales that covered aspects such as communication, convenience, and overall experience with telemedicine services. Qualitative interviews allowed patients to express their experiences in greater detail, providing insight into factors that influence satisfaction levels.

Healthcare provider satisfaction: The satisfaction of healthcare providers was assessed using similar methodologies. The surveys assessed providers' perceptions of the impact of telemedicine on their workflow, effectiveness in patient care, and overall satisfaction with technology. The interviews delved deeper into the nuances of provider experiences, uncovering challenges, facilitators, and suggestions for improvement.

Accessibility of healthcare services: Accessibility was evaluated through quantitative and qualitative measures. Geographic and logistic barriers to healthcare access were assessed using patient-reported data. In addition, the study explored changes in the frequency and ease of healthcare service utilization, providing a nuanced understanding of how telemedicine and remote monitoring influenced the accessibility of healthcare services.

Data analysis

The study data were subjected to thorough statistical analyses using IBM SPSS Version 23 software (IBM Corp., Armonk, NY). Appropriate tests, including t-tests and ANOVA for primary outcomes, such as patient health improvements and savings in healthcare costs, were conducted with a predefined significance level (p = 0.05). Subgroup analyses using tests, such as the chi-square or Fisher's exact test, were performed where applicable, applying the same significance level to evaluate specific demographic or clinical subpopulations.

## Results

Demographics

The demographic characteristics of the 186 participants were diverse, representing a broad age range, gender distribution, socioeconomic backgrounds, and geographic locations. The majority of the participants were in the age group 31-50, with a nearly equal distribution between male and female participants. Socioeconomic status was fairly balanced, and the sample included participants from urban, suburban, and rural areas (Table 1).

**Table 1:** Demographic characteristics of the 186 participants

Demographic characteristic	Frequency	Percentage (%)
Age group		
- 18-30 years	40	21.5
- 31-50 years	75	40.3
- 51-70 years	45	24.2
- 71+ years	26	14.0
Gender		
- Male	90	48.4
- Female	96	51.6
Socioeconomic status		
- Low income	30	16.1
- Middle income	110	59.1
- High income	46	24.8
Geographic location		
- Urban	120	64.5
- Suburban	45	24.2
- Rural	21	11.3

Patient health improvements

The mean value of disease-specific markers decreased significantly from 12,000 (SD ± 1,500) before intervention to 11,000 (SD ± 1,800) after intervention (p = 0.002), indicating an improvement in disease management. The severity of the symptoms showed a substantial reduction from 3,500 (SD ± 1,200) to 2,000 (SD ± 800) after the intervention (p < 0.001), demonstrating a positive impact on patient well-being. Participants experienced a significant improvement in overall health status, with a mean score increasing from 7,200 (SD ± 1,500) to 8,500 (SD ± 1,000) after intervention (p < 0.001) (Table 2).

**Table 2:** Outcomes related to patient health improvements

Patient health improvement metrics	Pre-intervention mean (SD)	Post-intervention mean (SD)	p-value
Disease-specific markers	₹12,000 (₹1,500)	₹11,000 (₹1,800)	0.002
Symptom severity	₹3,500 (₹1,200)	₹2,000 (₹800)	<0.001
Overall health status	₹7,200 (₹1,500)	₹8,500 (₹1,000)	<0.001

Healthcare cost savings

The implementation of telemedicine and remote monitoring resulted in a substantial reduction in direct healthcare costs, with a mean decrease from 25,000 (SD ± 8,000) to 12,000 (SD ± 4,000) after the intervention (p < 0.001).

Indirect costs, including productivity loss, also decreased significantly from 10,000 (SD ± 5,000) to 5,000 (SD ± 2,000) after intervention (p = 0.004), indicating economic benefits (Table 3).

**Table 3:** Healthcare cost savings

Cost category	Pre-intervention mean (SD)	Post-intervention mean (SD)	p-value
Direct costs (e.g., hospital visits)	₹25,000 (₹8,000)	₹12,000 (₹4,000)	<0.001
Indirect costs (e.g., productivity loss)	₹10,000 (₹5,000)	₹5,000 (₹2,000)	0.004

Patient and healthcare provider satisfaction

Patient satisfaction with communication improved significantly from 80% to 95% after the intervention (p < 0.001), indicating improved interaction between patients and healthcare providers. Satisfaction with the convenience of healthcare services increased from 75% to 90% after intervention (p < 0.001), suggesting improved accessibility.

The overall healthcare experience showed a positive change, with satisfaction increasing from 85% to 92% after intervention (p = 0.012). Healthcare providers reported higher satisfaction levels, increasing from 80% to 95% after the intervention (p < 0.001), indicating positive perceptions of telemedicine (Table 4).

**Table 4:** Patient and healthcare provider satisfaction

Satisfaction measure	Pre-intervention (%)	Post-intervention (%)	p-value
Patient communication	80	95	<0.001
Convenience of services	75	90	<0.001
Overall experience	85	92	0.012
Healthcare provider satisfaction	80	95	<0.001

Accessibility of healthcare services

Telemedicine contributed to a significant reduction in geographic barriers, with accessibility increasing from 65% to 90% post-intervention (p < 0.001). The frequency of healthcare utilization decreased from 2.5 to 1.5 after intervention (p < 0.001), indicating that healthcare services became more efficient and streamlined (Table 5).

**Table 5:** Accessibility of healthcare services

Accessibility metrics	Pre-intervention (%)	Post-intervention (%)	p-value
Reduction in geographic barriers	65	90	<0.001
Frequency of healthcare utilization	2.5	1.5	<0.001

## Discussion

Telemedicine and remote patient monitoring have emerged as transformative technologies in healthcare, offering innovative solutions to address various challenges in healthcare delivery.

Demographic and participant characteristics

The diverse demographic profile of the participants, including the distribution between age groups and gender, ensures a representative sample, acknowledging that the efficacy of telemedicine can vary between different populations [8]. Furthermore, the inclusion of participants from various socioeconomic backgrounds and geographic locations reflects the potential for widespread applicability of telemedicine technologies.

Patient health improvements

The observed improvements in disease-specific markers, severity of symptoms, and overall health status corroborate previous research indicating the positive impact of telemedicine on the management of chronic diseases [9]. Reductions in disease-specific markers, such as blood glucose levels or blood pressure, signify the potential for telemedicine to contribute to better health outcomes [10]. The substantial decrease in the severity of the symptoms aligns with studies that demonstrate the effectiveness of remote monitoring in the early detection and intervention of symptoms [11]. In general, these findings support the notion that telemedicine can improve patient health by facilitating continuous monitoring and early intervention, particularly in the management of chronic diseases [12].

Healthcare cost savings

Substantial reduction in direct and indirect healthcare costs is consistent with a growing body of evidence supporting the cost-effectiveness of telemedicine [13]. The decreased frequency of hospital visits and the associated direct costs echo the findings of studies on telemedicine interventions in chronic care management [14]. Furthermore, the observed decrease in indirect costs related to productivity loss is consistent with the economic benefits attributed to reduced travel time and missed workdays associated with telemedicine consultations [15]. These cost savings underscore the potential of telemedicine to address the economic burdens on both patients and healthcare systems [16].

Patient and healthcare provider satisfaction

The improvements in patient communication, convenience of services, and overall experience resonate with the existing literature that emphasizes the importance of patient-centered care in telemedicine [17]. The positive change in patient satisfaction with communication aligns with studies that highlight the role of effective communication in improving patient engagement and adherence to treatment plans [18]. Increased satisfaction with the convenience of services is consistent with the literature that emphasizes the flexibility and accessibility offered by telemedicine [19]. Patient satisfaction with healthcare providers is crucial for the successful integration of telemedicine into clinical practice [20]. The positive feedback from healthcare providers in this study supports findings that suggest telemedicine can improve healthcare delivery from the provider's perspective, fostering positive attitudes toward technology adoption [21].

Accessibility of healthcare services

The significant reduction in geographic barriers and the subsequent increase in healthcare access is a key outcome of this study, aligning with the overarching goal of telemedicine to overcome spatial limitations in healthcare delivery [22]. The findings emphasize the potential of telemedicine to address healthcare disparities by improving access for people living in remote or underserved areas [23]. The decrease in the frequency of healthcare utilization suggests that telemedicine not only improves accessibility but also streamlines healthcare delivery, minimizing unnecessary visits and optimizing resource allocation [2].

Overall, the results of this study align with and contribute to the broader body of literature on telemedicine and remote patient monitoring. Existing studies have shown the effectiveness of telemedicine in managing chronic diseases, improving patient outcomes, and reducing healthcare costs [24]. In addition, the literature on patient and provider satisfaction underscores the importance of user experience in the successful implementation and adoption of telemedicine technologies [25]. The observed reduction in geographic barriers is consistent with studies highlighting the potential of telemedicine to bridge gaps in healthcare access, particularly for individuals in rural or underserved areas [2, 19].

## Conclusions

In conclusion, this study contributes to the growing body of evidence supporting the positive impact of telemedicine and remote patient monitoring on healthcare delivery. The findings suggest that these technologies hold promise in improving patient health outcomes, reducing healthcare costs, enhancing patient and provider satisfaction, and addressing barriers to healthcare access. As technology continues to advance and healthcare systems evolve, the integration of telemedicine is poised to play an increasingly vital role in shaping the future of healthcare delivery.
